# Clostridioides difficile Infection: A Clinical Review of Pathogenesis, Clinical Considerations, and Treatment Strategies

**DOI:** 10.7759/cureus.51167

**Published:** 2023-12-27

**Authors:** Evan S Sinnathamby, Joseph W Mason, Chelsi J Flanagan, Nathan Z Pearl, Caroline R Burroughs, Audrey J De Witt, Danielle M Wenger, Vincent G Klapper, Shahab Ahmadzadeh, Giustino Varrassi, Sahar Shekoohi, Alan D Kaye

**Affiliations:** 1 School of Medicine, Louisiana State University Health Sciences Center (LSUHSC) New Orleans, New Orleans, USA; 2 School of Medicine, University of the Incarnate Word School of Osteopathic Medicine, San Antonio, USA; 3 School of Medicine, Louisiana State University Health Shreveport, Shreveport, USA; 4 Department of Medicine, University of Arizona College of Medicine - Phoenix, Phoenix, USA; 5 Department of Internal Medicine, Louisiana State University Health Shreveport, Shreveport, USA; 6 Department of Anesthesiology, Louisiana State University Health Shreveport, Shreveport, USA; 7 Department of Pain Medicine, Paolo Procacci Foundation, Rome, ITA

**Keywords:** intravenous immunoglobulin, fecal transplant, ileostomy, cholestyramine, endoscopy, nosocomial, diarrhea, colitis

## Abstract

Background: *Clostridioides difficile* infection (CDI) is a common nosocomial infection. Risk factors for developing CDI include prior hospitalization, being older than 65 years old, antibiotic use, and chronic disease. It is linked with diarrhea and colitis and can vary in severity. It is a major cause of increased morbidity and mortality among hospitalized patients. However, community-acquired CDI is also increasing. Proper diagnosis and determination of severity are crucial for the treatment of CDI. Depending on how severe the CDI is, the patient may endorse different symptoms and physical exam findings. The severity of CDI will determine how aggressively it is treated.

Management and treatment: Laboratory studies can be helpful in the diagnosis of CDI. In this regard, common labs include complete blood count, stool assays, and, in certain cases, radiography and endoscopy. Mild-to-moderate colitis is treated with antibiotics, but severe colitis requires a different approach, which may include surgery. Several alternative therapies for CDI exist and have shown promising results. This review will touch upon these therapies, which include fecal transplants, intravenous immunoglobulin, and the use of cholestyramine and tigecycline.

Conclusion: Prevention of CDI can be achieved by proper hygiene, vaccinations, and detecting the infection early. Proper hygiene is indeed noted to be one of the best ways to prevent CDI in the hospital setting. Overprescribing antibiotics is also another huge reason why CDI occurs. Proper prescription of antibiotics can also help reduce the chances of acquiring CDI.

## Introduction and background

*Clostridioides difficile* is a gram-positive bacillus spore-forming obligate anaerobe. It is commonly found in soil, water, and human and animal feces. It grows best at 37°C and is transmitted via the fecal-oral route. There are two forms of the organism: a dormant spore form that is resistant to medication and a vegetative form that can produce toxins and is susceptible to the activity of some medication [1]. *C. difficile* infections (CDIs) are known to be one of the most common nosocomial (hospital-acquired) infections and frequently increase morbidity and mortality in adult hospital patients [2]. However, CDI is being diagnosed at an increasing rate among younger patients in the community. There are around 450,000 people infected by *C. difficile* annually in the United States, with 14,000 associated deaths due to CDI [2]. Once the normal microbiota of the gut has been disrupted (frequently by antibiotic therapy), *C. difficile* can colonize the intestinal tract unopposed. Normal gut microbiota evolves with the host over time and plays a key role in the metabolism of nutrients, especially lipids [3]. Once *C. difficile* can grow uninhibited, colitis can form. Pseudomembranous colitis may also form, but it should be noted that pseudomembrane formation does not always occur in *C. difficile* colitis (CDC). Symptoms occur when the bacterium produces toxins that cause diarrhea and inflammation of the colon [3]. Specifically, *C. difficile* produces two toxins, toxin A (TcdA) and toxin B (TcdB) [4,5]. There is another toxin that has been identified in isolated species called a binary toxin, but its role in the pathophysiology of CDI has yet to be elucidated [6]. Toxin A uses a carbohydrate receptor to facilitate the internalization of itself and toxin B [7]. Both toxins cause the inactivation of several proteins in the colonocytes, which results in damage to tight junctions and colitis [8]. More than half of patients who are hospitalized will end up being prescribed antibiotics during their hospital stay [9]. About 30-50% of these patients are given antibiotics inappropriately [9]. Antibiotic misuse is thought to be a major reason why patients develop CDI in hospital settings. Damage to the gut microbiome by antibiotics and other drugs can allow *C. difficile* to proliferate in the gut unchallenged. All antibiotics are thought to increase the risk of developing CDI, but clindamycin, fluoroquinolones, and third-generation cephalosporins have been major causes of nosocomial CDI in the literature [10]. Prior hospitalization, age greater than 65 years old, and antibiotic use are the three major risk factors for developing CDI [11]. There are several comorbid conditions that contribute to the risk of mortality of CDI, like cognitive impairment and liver, renal, and ischemic heart diseases [12].

CDI usually presents with moderate-to-severe diarrhea; however, asymptomatic colonization is possible [13]. Furthermore, splenic access is an extremely rare extra-intestinal complication of CDI. Therefore, it is crucial to perform abdominal imaging in patients presenting with an acute abdomen to rule out toxic megacolon and splenic abscess [14]. Patients with CDI can be categorized as having a moderate, mild, or severe case. Determining the severity of the disease is important in the management of CDI [1]. Treatment can range from antibiotic treatment to surgery. This article will discuss how CDI presents clinically, how it is typically managed, what some alternative therapies are, and how to prevent both primary and secondary CDI. This review may help clinicians in confidently diagnosing and treating this disease in a timely manner.

## Review

Clinical presentation

History and Physical Exam Findings

When evaluating a patient suspected of *C. difficile*, it is important to focus the history on identifying offending medications such as antibiotics and high-risk patient features like older age or patients with an underlying chronic condition [15]. *C. difficile* can also manifest in a spectrum of diseases, from asymptomatic to toxic megacolon [16-18]. Additionally, the history should focus on establishing how severe the CDI is and whether this is a recurrent infection [18]. Severe CDI is defined by the appearance of serum albumin <3 g/dL plus a white blood cell (WBC) count of >15,000 cells/mm cube or abdominal tenderness [19]. All patients suspected of CDI require a thorough abdominal examination, basic laboratory testing, and potential radiographic evaluation [15]. This indication for further advanced testing depends on the patient's clinical manifestations [18]. If a patient is determined to have mild-to-moderate colitis, they usually endorse diarrhea without signs of systemic infection and typically have fewer laboratory derangements [16,18]. Patients with mild-to-moderate colitis are also more hemodynamically stable [16,17]. Fever, abdominal cramping, and discomfort may develop in these patients. Diarrhea with mucus and occult blood can be present, but frank hematochezia and melena usually are not [16,18]. Most physical exam findings in a person with CDI consist of lower abdominal pain. A thorough abdominal exam may be benign or demonstrate slight tenderness to palpitation [15]. The patient should not exhibit guarding, peritonitis, or rebound tenderness.

If a patient is determined to have severe colitis (e.g., fulminate colitis), they may exhibit diarrhea with strong evidence of systemic illness and end-organ dysfunction [17,18,20]. Risk factors for acquiring severe colitis include a history of chronic obstructive pulmonary disease (COPD), a history of renal failure, and infection with the hypervirulent strain B1/NAP1/027 [17,18,20]. Patients with fulminate colitis may also have hypotension and shock with concerns of bowel distension [18]. Patients may have a history of mild-to-moderate colitis, a recent history of abdominal surgery, or IBD. It is important to note that these patients may present with ileus and have signs and symptoms of a bowel obstruction rather than diarrhea. Patients with severe colitis have a greater chance of being hemodynamically unstable and should be given antibiotics, supportive care, resuscitations, and consideration for intensive care unit (ICU) admission if complications arise [16-18,21]. Typically, patients present febrile and endorse severe abdominal pain and distension. Commonly, patients may have exam findings of hypovolemia and present with reduced skin turgor or dry mucus membranes [16-18,20,21]. Patients may exhibit guarding, rebound tenderness, and peritonitis, which all raise suspicion for impending perforation of the colon [15].

Laboratory Data

Patients who have mild-to-moderate colitis should have a WBC count of 15,000 cells/mL or less, and they should also have a normal serum creatine of 1.5 mg/dL or less [17,18]. The antigen test for *C. difficile* should be positive as well. Diagnostic tests should be run on patients with significant diarrhea [22]. Diagnostic tests include nucleic acid amplification testing (NAAT), enzyme immunoassay (EIA), cell culture cytotoxicity assay, and selective anaerobic cultures. The 2017 Infectious Diseases Society of America (IDSA)/Society for Healthcare Epidemiology of America (SHEA) guidelines recommend testing patients with unexplained new-onset diarrhea with three or more unformed stools in 24 hours [4]. It should be noted that clinical judgment is still the strongest determinant in identifying a patient who is experiencing a symptomatic infection [4]. The highly sensitive gold standard for testing for detecting infection is a toxigenic stool culture or cell cytotoxicity neutralization assays, but these modalities are impractical outside of research settings [4]. The EIAs to detect toxins A+B provide rapid results with high specificity, but sensitivity is often impacted by sample handling [4]. NAAT offers similar sensitivity to toxigenic culture but can detect the presence of gene encoding toxin, which can confirm the presence of a toxin-producing strain, but not whether the toxin is being elaborated by the organism [4]. Glutamate dehydrogenase (GDH) is produced by both toxin-producing and non-toxin-producing strains. GDH EIA is extremely sensitive and functions as a screening tool with great negative predictive value [4]. If GDH EIA is positive, additional testing must be done to confirm the presence of a toxigenic strain [4]. Typically, EIA for GHD is performed first. An EIA testing for toxins A and B should also be performed. It should be noted that EIA for toxins A and B have a high false-negative rate due to the large amount of toxin needed for a positive test [23-25]. When discordant results are obtained, NAAT may be useful in detecting genes specific to toxic strains. It is very sensitive, and results usually come back within an hour. However, NAAT can lead to overdiagnosing and overtreating patients as it only checks for toxin genes and not the active production of toxins [26]. If an abdominal radiograph or computed tomography (CT) scan is obtained in patients with criteria for mild-to-moderate colitis, the findings would be generally benign and nonspecific, possibly showing signs of colonic inflammation [15]. Typically, radiography is not warranted in these patients. Laboratory data in a person with severe colitis may include a WBC count of >15,000 cells/mL and evidence of end-organ damage with creatine >1.5 mg/dL or greater than 1.5 times the patients' baseline creatine [18]. Patients may have a positive stool antigen test for *C. difficile* and may present with signs of hypovolemia [14]. Radiography of the abdomen and pelvis is usually warranted in patients with severe colitis. Plain abdominal X-rays may show dilated bowels as well as classic signs of bowel edema and inflammation called "target signs" and "thumb printing" [15]. CT scanning may show an "accordion sign," indicating pseudomembranous formation. Endoscopy can be performed in patients where ileus is present or alternative diagnoses may be suspected. Endoscopy may show pseudomembrane formation and patchy white/yellow plaques that may be continuous or patchy [27]. The absence of pseudomembranes does not rule out CDI. Conversely, the presence of pseudomembranes is not exclusive to CDI as other disease processes also can cause the formation of pseudomembranes, like IBD, cytomegalovirus, and *Entamoeba histolytica* infection [25].

Differential Diagnosis

*C. difficile* must be distinguished from other causes of diarrhea. Many causes of antibiotic-associated diarrhea are caused by osmotic mechanisms and not CDI. However, if colitis is present with diarrhea, it is almost always CDI. Acute abdomen caused by CDI may be present and can mimic small bowel ileus, Ogilvie's syndrome, volvulus, or ischemia [28]. Other antibiotic-associated diarrhea causes include *Staphylococcus aureus*, *Klebsiella oxytoca*, *Clostridium perfringens*, and *Salmonella* spp. Patients will often present with rectal symptoms that mimic IBD, like rectal pain, bloody discharge, and urgency [29]. Stool testing and culture are crucial for determining the diagnosis [29]. Noninfectious causes of diarrhea, like irritable bowel syndrome, IBD, celiac disease, and microscopic colitis, should also be considered.

Management

Patients with CDI are categorized based on the severity of their disease, grouped collectively as non-fulminant CDI and fulminant CDI (FCDI). Guidelines define FCDI as severe infection (WBC count ≥15,000 cells/mm^3^ or serum creatinine ≥1.5 mg/dL) along with hypotension, shock, ileus, or megacolon, requiring an ICU admission [30]. This section describes the management of non-fulminant CDI, differentiating primary vs. recurrent infection, along with the changes in management if a patient progresses to FCDI. For patients with primary infection of CDI, metronidazole should be considered over vancomycin or fidaxomicin for only the mildest cases [31]. Vancomycin and fidaxomicin are the preferred antibiotics due to metronidazole's poor pharmacokinetics in the intestinal system. The recommended dose regimen for metronidazole is 500 mg three times daily for 10 days, either per os (PO) or intravenous (IV), while the dose regimen for oral vancomycin is 125 mg four times daily (QID) for 10-14 days [31]. Higher doses of vancomycin are used in severe cases of colitis up to 500 mg QID. Fidaxomicin is a macrocyclic antibiotic approved in 2011 that has little intestinal absorption, leading to high levels within the colon. Because of this, fidaxomicin has shown comparable cure rates to vancomycin and is associated with lower rates of recurrence when compared to a 10-day regimen of vancomycin for non-NAP1/ribotype 027 strains [32,33]. Fidaxomicin, an expensive brand-name drug that may cause nausea, vomiting, and fatigue, has also been shown to have lower rates of new-onset colonization of vancomycin-resistant *Enterococcus* (VRE) and *Candida* species following CDI [34]. There is some evidence to suggest that treatment with fidaxomicin results in the reduced recurrence of CDI compared to treatment with vancomycin [35].

Because of this, IDSA guidelines recommend treatment of initial and recurrent CDIs with fidaxomicin rather than vancomycin [36]. The recommended dosing regimen for fidaxomicin is 200 mg PO twice daily for 10 days. In patients with recurrent CDI, managing the first recurrence involves discontinuing non-CDI antibiotics. Suppose vancomycin or fidaxomicin were used to treat the primary infection. In that case, these medications are still indicated for use in the primary recurrence, as the recurrence is not due to the development of antimicrobial resistance [31]. The dosing regimen for both is the same as the treatment of a primary infection. Fidaxomicin again showed a more favorable profile in reducing the risk of subsequent recurrence following treatment [37]. Treatment for subsequent recurrent infections uses the same dose regimen of vancomycin, followed by six to seven weeks of a tapered dose or pulse strategy dose of vancomycin to inhibit vegetative *C. difficile* cells from overgrowing while simultaneously allowing the microbiota to regrow and recolonize the colon [31]. The tapered dose regimen of vancomycin is as follows: week 1: 125 mg QID, week 2: 125 mg twice daily, week 3: 125 mg once daily, week 4: 125 mg every other day (four doses given), and weeks 5 and 6: 125 mg every three days [31]. The pulse strategy dosing regimen is simpler, with the patient taking 125 mg every two days or 500 mg every three days for three weeks [34].

For patients with FCDI, the risk of recurrence becomes secondary to ensuring the survival of the patient. For non-fulminant disease, antibiotic dosing considers the patient's microbiota and attempts to maintain it while combating the overgrowth of *C. difficile*. However, in FCDI, treatment is much more aggressive, using higher doses and combination therapy [30]. The pharmacokinetic profile of a patient with FCDI is different from that of a non-fulminant patient and can lead to further complications in the delivery of treatment options. Dehydration due to diarrhea can lead to hypovolemic shock, causing hypoperfusion of the gut and thereby decreasing gut absorption in the small intestine [38]. Furthermore, gastrointestinal hypermobility, caused by diarrhea, can reduce the contact time in both the small and large intestines, lowering the effectiveness of the antibiotics [38]. The formation of an ileus can also slow or prevent an antibiotic from getting to the site of infection [30]. Treatment guidelines aim to alter antibiotic therapy in the treatment of FDCI to maximize both the dosage delivered to the site of infection and the amount of time the drug can act upon the infected tissue. Oral vancomycin is recommended at an increased dose (500 mg) QID, combined with IV metronidazole and vancomycin as a retention enema [38,39]. A retention enema is further indicated in a patient with an ileus to ensure enough antibiotics are delivered to the site of infection. The dosing of the vancomycin enema is disputed in the literature, but recent American College of Gastroenterology (ACG) guidelines suggest 500 mg of vancomycin in 100 mL of saline [4]. While fidaxomicin has shown a favorable profile in the treatment of non-fulminant CDI, there is a lack of literature that supports its use in the setting of FCDI [33]. In severe cases of CDI, such as FCDI, surgical intervention may be needed to ensure the survival of the patient. The formation of an ileus is an ominous sign of worsening infection and increases the risk of bowel necrosis, necessitating surgery as a life-saving measure [40]. Special care must be taken so that any surgical intervention is performed while the patient is stable enough to survive the operation without major complications. Current guidelines recommend two surgical interventions: a total abdominal colectomy (TAC) with end ileostomy or diverting loop ileostomy (LI) intraoperative colonic lavage and anterograde vancomycin flushes [30]. LI has been shown to have a similar mortality benefit to TAC and has several benefits to the patient, being done laparoscopically [30,41]. Patients can retain their colon with LI, along with easier management of reanastamosis [16,40,42]. Factors to consider when determining whether surgical intervention is warranted include failure of medical therapy, advanced age, septic shock, requiring vasopressors, end-organ failure, peritonitis, toxic megacolon, and colonic rupture [30]. Biomarkers can also be used to determine eligibility, such as WBC count ≥50,000 cells/mm^3^, lactate ≥5.0 mmol/L, and albumin <1.5 g/dL [30]. However, clinical judgment is key in determining which patients would benefit from surgery, weighing potential risks and benefits from the procedure [30].

Fecal microbiota transplantation (FMT)

FMT is a procedure in which fecal matter collected from a donor is mixed with saline, strained, and then placed into a patient via colonoscopy to cure the existing CDI [43]. Before its use for the eradication of CDI, FMT was first demonstrated as a tangible treatment option for patients with pseudomembranous colitis in 1958 [44]. Because patients with CDI have been found to have reduced gastrointestinal bacterial diversity, it is hypothesized that infusing feces from donor patients who have a good bacterial population can help to protect patients who have had their bacterial flora diminished secondary to CDI [45]. Moreover, published reports have shown that the use of FMT in certain patient populations increased cure rates by up to 92% [46]. Although FMT has demonstrated success in the treatment of CDI, its use is limited by the reluctance of patients to undergo the procedure and the hesitancy of physicians to choose this treatment option early in a patient's clinical course [43]. Additionally, there are concerns that FMT could propagate the spread of human immunodeficiency virus (HIV) and hepatitis or change the microbiome in the donor patient, leading to increased susceptibility to conditions like obesity or autoimmune disorders, but donors are screened very heavily before being able to donate fecal microbiota [47]. In 2022, REBYOTA (Ferring Pharmaceuticals Inc., Saint-Prex, Switzerland), a novel first-in-class microbiota live biotherapeutic, was approved for the prevention of recurrence of CDI in individuals 18 years or older following the antibiotic treatment of CDI [48].

Alternative therapies

Aside from the previously described mainstay therapies for CDI, several forms of alternative therapies also exist. Intravenous immunoglobulin (IVIG) has also been studied as a potential treatment for CDI. The severity and duration of symptoms caused by CDIs are primarily determined by the magnitude of the immune response to the bacteria [49]. The first report on the successful use of IVIG in the treatment of CDI was published in 1991; since then, IVIG has been utilized in an off-label fashion for this purpose [50]. The use of IVIG is currently not in published guidelines for the treatment of CDI. The two main mediators of CDI are toxin A and toxin B, which can cause a variety of clinical presentations, ranging from mild diarrhea to serious fulminant colitis [27,51]. Since its initial publication, IVIG has demonstrated its ability to neutralize toxin A and toxin B. Patients infected with *C. difficile* receive pooled immunoglobulin from several donors who express high anti-toxin A and anti-toxin B antibody serum titers [50]. Reports published more recently demonstrate that the severity of *C. difficile* disease correlates more with anti-toxin A levels, with anti-toxin B antibodies playing an adjunctive role in establishing immunity against CDI [52,53]. In 2010, Abougergi et al. performed a large retrospective observational study evaluating patients with severe CDC treated with IVIG [54]. In this study, 21 of 1230 patients with CDC were treated with IVIG infusion after conventional treatment with antibiotics for about eight days, with nine of the 21 patients surviving their hospitalization with complete colitis resolution. For survivors, symptoms resolved after about 10 days. The authors of this study concluded that the benefit of IVIG appears to depend on the extent of systemic involvement and recommended that more, larger-scale studies are required before using IVIG as routine treatment for severe CDC.

Interestingly, cholestyramine is an anion-binding resin, a medication that binds to negatively charged molecules, that has demonstrated its ability to prevent CDI in patients on ceftriaxone antibiotic therapy [55]. Cholestyramine is thought to bind toxins A and B, preventing their entry into host cells [56]. Puri et al. conducted a pilot study that observed the effects of concomitant cholestyramine therapy with ceftriaxone therapy in patients treated with Lyme disease [55]. In this study, 46 patients were treated with 2 grams of ceftriaxone daily and 4 grams of cholestyramine daily; only three out of the 46 participants developed *C. difficile*-associated diarrhea. They found that the incidence of *C. difficile*-associated diarrhea in this cohort was 6.5%, significantly lower than published results of 23-35% of patients receiving the same antibiotic therapy with no additional cholestyramine. The authors concluded that the results of their study were promising but that further investigation is necessary. It should be noted that cholestyramine therapy is not currently in IDSA guidelines. Another anion-binding resin, tolevamer, was tested in a multinational, randomized controlled trial against vancomycin and metronidazole. In this study, 563 patients with CDI received a 2:1:1 ratio of 9 g of oral tolevamer, followed by 3 g every eight hours for 14 days [57]. Results showed that tolevamer was clinically inferior to both vancomycin and metronidazole [57].

Currently, the ACG recommends the use of oral vancomycin and IV metronidazole as treatment for severe cases of CDI [58]. However, the number of cases of CDI that are refractory to this standard treatment is increasing due to the development of "hypervirulent" *C. difficile* strains that are resistant to this antibiotic treatment, creating a need for more novel antibiotic options [59]. In a retrospective observational cohort study, Gergely Szabo et al. examined the use of tigecycline for the treatment of severe CDI [60]. Out of the 395 patients who were hospitalized with severe CDI, the patients who were treated with tigecycline demonstrated significantly better outcomes of clinical cure and experienced less complicated disease course.

Prevention

Primary Prevention

Because *C. difficile* is one of the leading causes of hospital-acquired infectious diarrhea, proper prevention strategies are of high importance. Some strategies of primary prevention include sanitary control measures, proper antibiotic stewardship, and vaccine utilization. The Centers for Disease Control and Prevention (CDC) Emerging Infections Program (EIP) recently conducted population-based surveillance of CDIs from 10 cities in the United States from 2011 to 2017. The study reported an estimated 306,500 cases of healthcare-associated infections in 2011 compared to 235,700 cases in 2017. As for community-associated infections, there were 170,000 cases in 2011 and 226,400 cases in 2017 [61]. Despite the decrease in hospital-acquired CDIs, refined public sanitation efforts and biotechnology like vaccines remain valuable tools that could help decrease national infection rates. Sanitary control measures include quick identification of infected patients, proper isolation and documentation, thorough room disinfection, and use of personal protective equipment [62]. Hand hygiene remains a crucial aspect of preventing transmission. While alcohol-based hand washing may effectively reduce the vegetative form of *C. difficile*, soap and water are necessary to remove both vegetative bodies and spores [63]. Prolonged prior antibiotic use remains an important risk factor for CDI [62]. Antibiotics like cephalosporins, fluoroquinolones, clindamycin, and penicillins like amoxicillin-clavulanate may increase the risk of CDI [64]. A quality improvement study revealed the benefit of implementing antibiotic stewardship programs in 402 hospitals. In one year, antibiotic use decreased by 30 days of antibiotic therapy per 1000 patient days, and the incidence rate of hospital-acquired CDI decreased by 19.5% [65]. A phase II randomized controlled trial examined a candidate toxoid vaccine against *C. difficile* toxins A and B. The high dose with adjuvant formulation administered at 0-7-30 days had the greatest antibody response at day 180 and was selected for phase III clinical trials [66]. The phase III *Clostridium difficile* Vaccine Efficacy Trial (CLOVER) examines the candidate vaccine use in patients over 50 years old. Initial results have proved promising. However, the activity study has not been completed and submitted to a peer-reviewed journal [62].

Secondary Prevention

In addition to the antibiotic regimes discussed above, other secondary prevention strategies, including early detection and monoclonal antibody treatments, can help prevent the severity and recurrence of CDIs. Early detection of *C. difficile* can help prevent the spread of the bacteria. The ACG currently recommends testing patients for *C. difficile* with ≥3 unformed stools in 24 hours [4]. The ACG testing algorithm aligns with the European Society of Clinical Microbiology and Infectious Diseases. They both currently suggest that laboratory diagnosis should involve a two-step approach. The initial screening test should consist of a nucleic acid amplification test detecting toxin genes or an EIA detecting GDH. The subsequent confirmational test should be an EIA detecting free toxins A and B [67].

Toxigenic strains of *C. difficile* produce toxins A and B, which are responsible for host damage. In 2016, the Food and Drug Administration (FDA) approved bezlotoxumab, a monoclonal antibody for toxin B, to prevent recurrent *C. difficile*. Two phase III randomized controlled trials, Macrocyclic Orally Administered Fidaxomicin in CDI Patients (MODIFY I) and Macrocyclic Orally Administered Fidaxomicin in the Treatment of *Clostridium difficile* Infection (MODIFY II), examined the use of monoclonal antibodies actoxumab (against toxin A) and bezlotoxumab in 2655 patients with recurring CDI. Groups received either bezlotoxumab alone, bezlotoxumab plus actoxumab, or a placebo. Actoxumab monotherapy was given in MODIFY I but was discontinued due to an increased rate of recurrent infections, adverse effects, and deaths. The bezlotoxumab alone and bezlotoxumab plus actoxumab groups had a lower recurrence rate than the placebo in both MODIFY I and MODIFY II. There was no significant difference in recurrence rate between bezlotoxumab monotherapy and bezlotoxumab combination therapy [68].

## Conclusions

CDI is one of the most common nosocomial infections in the United States. It increases morbidity and mortality in hospitalized patients. Antibiotics are a major cause of CDI in the hospital and can kill off the natural microbiota and allow *C. difficile* to grow without any competition. Infection with *C. difficile* can present as mild-to-severe colitis. It is important that clinicians distinguish the severity of CDI, as this will determine how it is treated. Stool EIAs, NAATs, CBCs, and radiography can aid in the diagnosis of CDI. Metronidazole given IV can be prescribed to treat mild cases of primary CDI. Oral vancomycin and fidaxomicin can also be used for more severe cases of primary CDI. Recurrent CDI may still be treated with vancomycin and fidaxomicin if these medications were used before. FCDI requires a more aggressive approach to treatment. Surgery may also be warranted with severe forms of CDI. Current guidelines recommend two surgical interventions: TAC with end ileostomy or diverting LI intraoperative colonic lavage and anterograde vancomycin flushes. Alternative therapies also exist for CDI. These include fecal microbiota transplants and neutralization of toxins A and B with IVIG, cholestyramine, and tigecycline. Sanitary control measures, vaccination, and antibiotic stewardship can reduce the chances of primary infection. Secondary infections can be reduced by early detection and monoclonal antibody treatment.
